# Preclinical and clinical evaluation of vancomycin plus delpazolid combination therapy for MRSA bacteremia: a multicenter, double-blinded, randomized, parallel design, phase IIa clinical trial

**DOI:** 10.1128/spectrum.03361-25

**Published:** 2026-02-18

**Authors:** Kyung-Hwa Park, Jongtak Jung, Sung Un Shin, Su Jin Jeong, Dae Won Park, Dong-Min Kim, Seong Jin Choi, Song Mi Moon, Jeong Su Park, Kyoung-Ho Song, Eu Suk Kim, Young Lag Cho, Seongman Bae, Hong Bin Kim, On behalf of the Korea INfectious Diseases (KIND) Study Group

**Affiliations:** 1 Department of Infectious Diseases, Chonnam National University Hospital, Chonnam National University Medical School 65417, Gwangju, Korea; 2 Division of Infectious Diseases, Department of Internal Medicine, Soonchunhyang University Seoul Hospital, Seoul, Korea; 3 Division of Infectious Diseases, Department of Internal Medicine and AIDS Research Institute, Yonsei University College of Medicine 37991, Seodaemun-gu, Korea; 4 Department of Internal Medicine, Korea University Ansan Hospital, Korea University College of Medicine, Seoul, Korea; 5 Department of Internal Medicine, Chosun University Hospital, Gwangju, Korea; 6 Department of Internal Medicine, Seoul National University Bundang Hospital, Seoul National University College of Medicine 65462, Seongnam, Korea; 7 Department of Laboratory Medicine, Seoul National University Bundang Hospital, Seoul National University College of Medicine 65462, Seongnam, Korea; 8 LigaChem Biosciences Inc., Daejeon, Republic of Korea; 9 Department of Internal Diseases, Asan Medical Center, University of Ulsan College of Medicine 65462, Seoul, Korea; Earlham Institute, Norwich, United Kingdom

**Keywords:** MRSA bacteremia, vancomycin, delpazolid, combination therapy, clinical trial

## Abstract

Methicillin-resistant *Staphylococcus aureus* (MRSA) bacteremia is a serious clinical challenge due to limited treatments and high mortality. We conducted both preclinical and clinical studies to assess the potential role of delpazolid in combination with vancomycin. Synergistic effects of delpazolid with vancomycin or daptomycin were evaluated *in vitro* using checkerboard and time-kill assays, and *in vivo* using a *Galleria mellonella* model. A multicenter, double-blind, randomized, Phase IIa trial was conducted at six Korean hospitals between 26 April 2022 and 18 March 2024. Patients with MRSA bacteremia were randomized 1:1 to receive vancomycin monotherapy or vancomycin plus delpazolid for 14 to 42 days. The primary outcome was overall cure at day 14 (microbiological clearance and symptom resolution). Secondary endpoints included safety, adverse events, and delpazolid pharmacokinetics. *In vitro* checkerboard assays showed no interaction between delpazolid and vancomycin or daptomycin, while time-kill assays revealed antagonism only when delpazolid was combined with vancomycin. In the *Galleria mellonella* model, combination therapy improved survival over monotherapy. In the clinical study, 40 patients were enrolled, 38 received ≥1 dose (safety set), and 34 (monotherapy: 19; combination: 15) were included in the full analysis set. On day 14, overall cure was 52.6% in the monotherapy and 60.0% in the combination group (*P* = 0.6675). Adverse event rates were similar across groups, with no significant safety concerns. In pharmacokinetic analyses, delpazolid showed favorable plasma levels when co-administered. These preliminary findings warrant further investigation in adequately powered trials to define the role of delpazolid plus vancomycin in the treatment of MRSA bacteremia.

**CLINICAL TRIALS:**

The study is registered with ClinicalTrial.gov as NCT05225558.

**IMPORTANCE:**

Methicillin-resistant *Staphylococcus aureus* (MRSA) bacteremia presents a serious clinical challenge due to limited treatments and high mortality. This study evaluated the potential role of delpazolid, an oral oxazolidinone, in combination with vancomycin for MRSA bacteremia. Preclinical studies demonstrated delpazolid’s antimicrobial activity comparable to vancomycin and daptomycin, and in the *Galleria mellonella* infection model, combination therapy significantly improved survival rates over monotherapy. The early-terminated Phase IIa clinical study showed that the combination regimen had an acceptable safety profile, with no apparent increase in adverse events compared to vancomycin monotherapy. While overall cure rates and bacteremia clearance were numerically higher in the combination group, these differences were not statistically significant. These preliminary findings underscore the need for larger, adequately powered clinical trials to clarify the clinical role of delpazolid combination therapy in MRSA infections.

## INTRODUCTION

*Staphylococcus aureus* bacteremia (SAB) is a major global healthcare concern associated with high mortality, morbidity, and economic burden (1, 2). Despite advances in antimicrobial therapy, persistent and recurrent infections remain significant challenges in SAB management. Methicillin-resistant *S. aureus* (MRSA) infections are particularly concerning, as they are linked to higher in-hospital mortality rates compared to methicillin-susceptible *S. aureus* (MSSA) (2).

Although several newer antibiotics with MRSA activity, such as daptomycin, linezolid, ceftaroline, and ceftobiprole, have been developed (3, 4), treatment options remain limited due to issues with accessibility, high costs, potential side effects, and lack of data from large-scale randomized controlled trials. Vancomycin remains the first-line therapy for MRSA bacteremia in most clinical settings; however, its efficacy is debated due to minimum inhibitory concentration (MIC) values approaching the susceptible breakpoint, challenges achieving adequate serum levels, heteroresistance, tolerance, and potential toxicity (5). Furthermore, vancomycin-intermediate *S. aureus* strains occasionally emerge, further complicating treatment (5). Linezolid is an alternative option, but it exhibits inter-individual pharmacokinetic variability and is associated with myelosuppression, lactic acidosis, and hepatic dysfunction (6). Additionally, recent reports indicate the emergence of linezolid- and daptomycin-resistant gram-positive cocci (7).

Delpazolid (LCB01-0371) is an oral oxazolidinone under development by LigaChem Biosciences (Daejeon, South Korea) for the treatment of MRSA, *Mycobacterium tuberculosis,* and refractory *Mycobacterium abscessus* complex (8). Preclinical studies have demonstrated its activity against gram-positive bacteria in both *in vitro* and *in vivo* models (8). Phase I clinical trials, including single and multiple ascending dose studies, have shown a favorable safety profile and a dose-proportional pharmacokinetic (PK) profile with oral formulations (9, 10). More recently, a Phase II trial for *M. tuberculosis* demonstrated bactericidal efficacy with lower toxicity than other oxazolidinones (11).

Building on these findings, we conducted a preclinical study to evaluate the *in vitro* efficacy and synergistic activity of delpazolid with standard anti-MRSA antibiotics, such as vancomycin and daptomycin, followed by *in vivo* evaluation using a *Galleria mellonella* infection model (12). Based on these preclinical results, Phase II clinical trial was conducted to evaluate the efficacy, safety, and pharmacokinetics of delpazolid in combination with vancomycin compared to vancomycin monotherapy for MRSA bacteremia.

## MATERIALS AND METHODS

### Preclinical study

#### *In vitro* antibiotic susceptibility and combination assays

The minimum inhibitory concentrations (MICs) of delpazolid, vancomycin, daptomycin, and linezolid against MRSA clinical isolates were previously determined using the broth microdilution method, following Clinical and Laboratory Standards Institute (CLSI) guidelines (13, 14). To evaluate the *in vitro* synergy of delpazolid with daptomycin or vancomycin, checkerboard and time-kill assays were performed. The checkerboard assay utilized *S. aureus* LAC (MRSA USA300) and ATCC 29213 (MSSA) strains. This assay was performed in 96-well plates using the broth microdilution method, with concentrations for each antibiotic ranging from 1/4× MIC to 16× MIC. The fractional inhibitory concentration (FIC) index (∑FIC) was calculated to classify interactions as synergistic (∑FIC ≤ 0.5), additive (∑FIC > 0.5 to ≤ 1), indifferent (∑FIC > 1 to ≤ 4), or antagonistic (∑FIC > 4).

For the time-kill assay, the LAC strain was tested using the macrodilution method with an initial inoculum of 5 × 10^5^ colony-forming units (CFU)/mL. Delpazolid was tested at 1×, 2×, and 4× MIC, while vancomycin and daptomycin were tested at 1× MIC, both alone and in combination with delpazolid. Synergy was evaluated by combining vancomycin or daptomycin (1× MIC) with delpazolid (2× and 4× MIC). Bacterial counts were quantified at multiple time points over 24 h. Synergy or antagonism was defined as a ≥2-log₁₀ CFU/mL increase or reduction in bacterial count at 24 h with the combination compared to the most effective single agent (15).

#### Galleria mellonella infection model

The *in vivo* antibacterial efficacy of delpazolid was evaluated using the *G. mellonella* infection model, following previously described methods (16). Healthy *G. mellonella* larvae were injected in the last left proleg with 10 µL of an MRSA LAC strain suspension (1 × 10^10^ CFU/mL). The infected larvae were divided into a total of 8 groups (*n* = 15 per group) conducted as two parallel experiments. The vancomycin combination experiment included: (1) control (phosphate buffered saline injection), (2) delpazolid (2 mg/kg), (3) vancomycin (2 mg/kg), and (4) delpazolid + vancomycin. The daptomycin combination experiment included: (1) control (phosphate buffered saline injection), (2) delpazolid (2 mg/kg), (3), daptomycin (8 mg/kg), and (4) delpazolid + daptomycin. These experiments were repeated independently three times. Larval survival was recorded at 24, 48, 72, and 96 h, and Kaplan–Meier survival analysis using the log-rank test was conducted for comparisons between the combination group and each monotherapy group in R software (version 4.3.3, R Foundation for Statistical Computing, Vienna, Austria).

All preclinical studies were conducted at the Central Microbiology Laboratory, Seoul National University Bundang Hospital.

### Clinical study

#### Study design

We conducted a Phase II, multicenter, double-blind, randomized, parallel-group trial (ClinicalTrials.gov identifier: NCT05225558) to evaluate adjunctive delpazolid in adults with MRSA bacteremia. The trial was conducted across six hospitals in South Korea. While the sample size was not formally calculated for statistical hypothesis testing, the target enrollment was 100 participants (50 in the study group and 50 in the control group). The study protocol was approved by the Institutional Review Board of Chonnam National University Hospital (CNUH-2021-417), Seoul National University Bundang Hospital (B-2111-722-001), Asan Medical Center (2021-1788), Yonsei National University Hospital (4-2023-0072), Chosun University Hospital (2023-01-009), and Korea University Ansan Hospital (2023AS0050). All participants provided written informed consent. LigaChem Biosciences (Daejeon, South Korea), the study sponsor, designed and conducted the trial in collaboration with the principal investigator and interpreted the study data alongside the authors. All biological analyses were performed in a blinded manner at independent laboratories.

#### Participants

Eligible patients were those who met the following criteria:

- Age ≥19 years
- At least one positive blood culture for MRSA confirmed within 96 h before randomization
- Initiation of empirical vancomycin treatment within 72 h prior to randomization
- Presence of clinical signs or symptoms of infection

Exclusion criteria were as follows: (i) polymicrobial bloodstream infection, (ii) receipt of empirical antibiotics for >96 h before randomization (except where vancomycin was initiated within 72 h), (iii) septic shock, (iv) severe immunosuppression (absolute neutrophil count <0.5 × 10⁹/L), or (v) expected mortality within 48 h due to MRSA bacteremia complications, as determined by predefined clinical indicators (e.g., refractory shock or multiorgan failure, or impending cardiac arrest, etc.) assessed by the investigator. Additional exclusion criteria included a body mass index of ≥35 kg/m² and inability to take oral medications. *S. aureus* identification and oxacillin susceptibility were confirmed at each institution following CLSI guideline. Written informed consent was obtained from all participants or their legal representatives (in cases of incapacity). Patients were recruited by the study team in collaboration with the hospital care team responsible for their in-hospital management.

#### Randomization and masking

After providing written informed consent, participants underwent screening tests and procedures. Eligible participants were randomly assigned in a 1:1 ratio to receive either delpazolid combined with vancomycin (combination group) or vancomycin with a matching placebo (monotherapy group). Placebo tablets were identical in appearance to delpazolid to ensure blinding. To ensure stratified block randomization at each clinical trial site, a statistician not directly involved in the trial generated the randomization code using SAS (version 9.4, SAS Institute Inc., Cary, NC, USA). Participants who met the inclusion/exclusion criteria were then assigned to a treatment group in the order of enrollment via an interactive web response system based on the randomization code.

Trial doctors, nurses, and hospital pharmacists involved in routine patient care were blinded to group allocation and treatment, while trial statisticians remained unmasked. Each trial center received participant-blinded treatment packs labeled only with a trial number containing either active delpazolid (400 mg tablets) or identical placebo tablets for the full treatment duration, according to the randomization list.

#### Procedures

Randomized participants received the assigned study drug for up to 42 days (minimum 14 days). Treatment included oral delpazolid 800 mg twice daily and an initial intravenous vancomycin dose of 15–20 mg/kg every 8–12 h (assuming MIC_broth microdilution (BMD)_ = 1 µg/mL). Subsequent vancomycin doses were adjusted to maintain an area under the concentration-time curve (AUC)/MIC_BMD_ ratio of 400–600. If the investigator determined that an alternative antibiotic was necessary for the treatment of MRSA bacteremia, vancomycin could be substituted with daptomycin after a minimum of 7 days of vancomycin administration. Upon discontinuation of vancomycin, daptomycin (6–10 mg/kg, intravenously, every 24 h) was initiated, and scheduled visits continued as planned. Participants were required to receive at least 14 days of intravenous vancomycin (or daptomycin, if switched) before transitioning to an oral antibiotic, excluding oxazolidinone-class drugs. If this switch met protocol criteria, study procedures continued as scheduled; otherwise, participants were withdrawn from the trial, excluded from the efficacy analysis, but retained in the safety analysis set.

Participants exited the trial 4 weeks after the end-of-treatment (EOT), with clinical assessments conducted on days 1, 3, 5, 7, and 14, followed by weekly assessments until EOT and a test-of-cure (TOC) visit. Blood cultures were obtained on days 1, 3, 5, and 7, then every 3 days until two consecutive negative MRSA results were documented. Additional cultures were performed at day 14 and at the EOT visit. Laboratory evaluations were performed on days 1, 7, 14, and at EOT, with the final TOC visit conducted in person.

#### Outcomes

Efficacy was primarily evaluated using the full analysis set (FAS), with supportive analyses performed on the per-protocol set (PPS). The primary efficacy endpoint was the proportion of participants achieving an overall cure within 14 days of treatment initiation (composite response rate), calculated as follows:


$$\left(\frac{number\;of\;participants\;with\;overall\;cure\;within\;14\;days}{number\;of\;participants\;in\;the\;efficacy\;analysis\;population}\right)\times 100,$$

Overall cure was defined as both clinical improvement and clearance of MRSA bacteremia, confirmed by two consecutive negative blood cultures. Key secondary efficacy endpoints included: (i) the proportion of participants achieving overall cure by the EOT visit; (ii) mortality due to MRSA bacteremia during the treatment period; (iii) the relapse rate of MRSA bacteremia before TOC visit, conducted 4 weeks post-EOT; (iv) the proportion of participants achieving clearance of MRSA bacteremia at days 3, 5, 7, 14, and EOT; (v) the proportion of participants with persistent MRSA bacteremia at days 3, 5, 7, and 14; (vi) the time (in days) to achieve clearance of MRSA bacteremia. Definitions of clinical and microbiological outcomes are detailed in Table S1.

Safety outcomes were evaluated using the safety analysis set. Assessments included: treatment-emergent adverse events (AEs), adverse drug reactions (ADRs), mortality due to MRSA bacteremia at the TOC visit, and the incidence of thrombocytopenia. Concomitant medication data were coded using the WHO Drug Dictionary 2024 and categorized by anatomical main group and preferred name. AEs and medical history were coded according to system organ class and preferred terms using the Medical Dictionary for Regulatory Activities version 27.0.

Pharmacokinetic (PK) modeling and analysis were conducted at Asan Medical Center, Republic of Korea, in accordance with a separate PK analysis plan. Delpazolid PK evaluation was performed using a two-compartmental model with NONMEM. The PK parameters assessed included: maximum plasma concentration (*C*_max_), AUC_last_, time to reach Cmax (*T*_max_), half-life (*T*_1/2_), and clearance. Serial blood samples were scheduled at six or more time points: between 30 min and 1 h and again between 2 and 8 h after administration of the investigational product on day 1 and again after reaching a steady state (after day 3 or later). All participants followed the same sampling schedule without randomization to alternative time points. The MICs of vancomycin, daptomycin, linezolid, and delpazolid against blood isolates from clinical trial patients were determined using the broth microdilution method at the central microbiology laboratory to evaluate drug susceptibility and resistance patterns.

#### Statistical analyses

**Fig 1:**
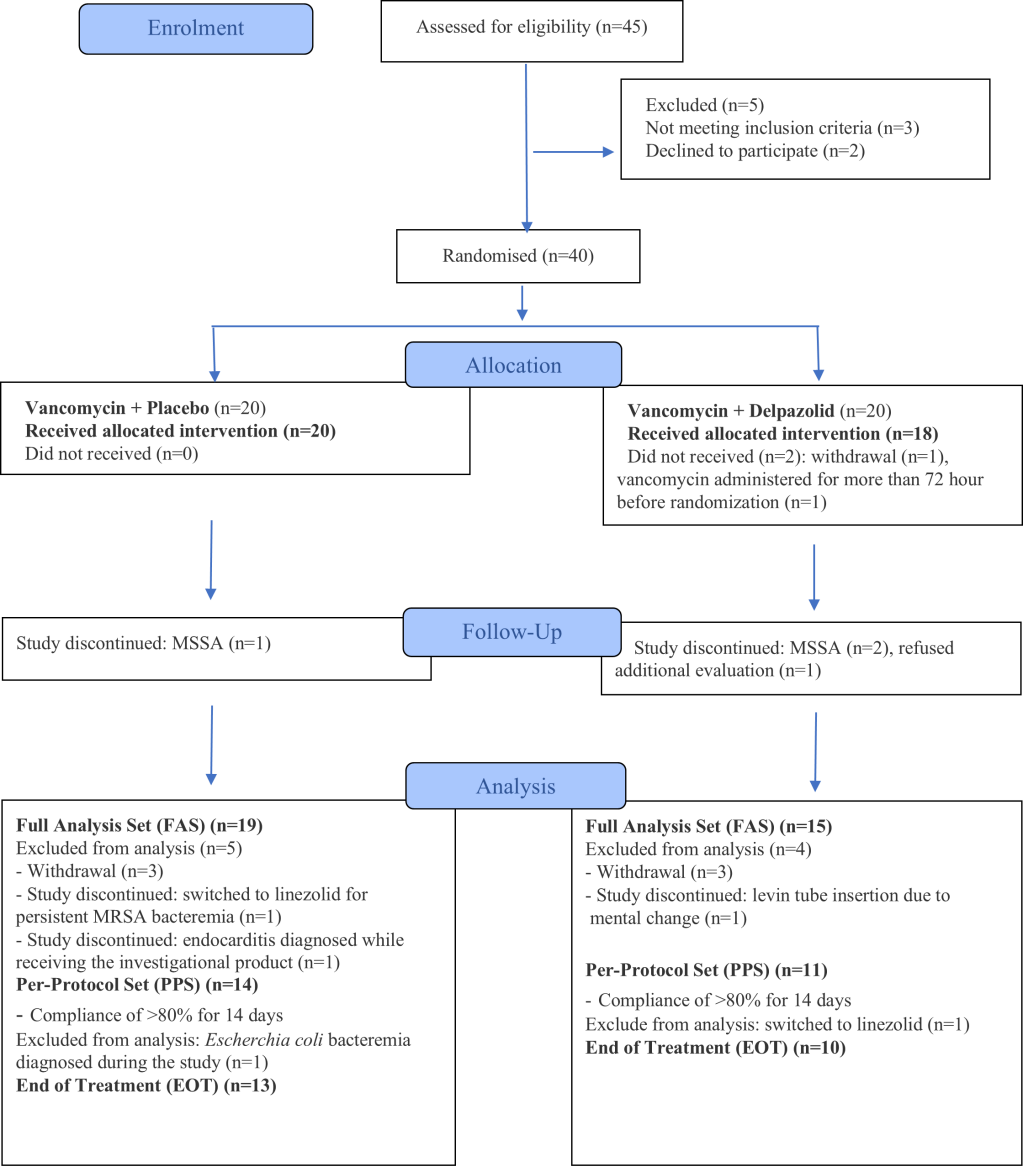
Disposition of study participants. Flowchart depicting the screening, randomization, and treatment of study participants. A total of 40 patients were enrolled in the study. Two patients did not receive the study drug and were excluded from further analysis. MSSA, methicillin-susceptible *Staphylococcus aureus.*

The study population included participants with confirmed MRSA bacteremia. Data were analyzed in three distinct analysis sets: the safety set, the FAS, and the PPS. The safety set included all randomized participants who received at least one dose of the study drug. The FAS comprised randomized participants who received at least one dose of the study drug, were confirmed to have MRSA, and were considered evaluable for efficacy. Participants in the FAS were analyzed according to their randomized treatment assignment. The PPS included participants from the FAS who received the study drug with ≥80% compliance during the first 14 days of treatment (Fig. 1). Safety outcomes were summarized and reported for the safety set, whereas primary and secondary efficacy analyses were conducted primarily on the FAS, with additional analyses performed on the PPS.

All statistical analyses were performed using SAS version 9.4 or later (SAS Institute Inc., Cary, NC, USA). Continuous variables were summarized using the number of participants, mean, standard deviation, median, minimum, and maximum. Categorical variables were reported as frequencies and percentages. A two-sided test was conducted at a 5% significance level. Differences in proportions between treatment groups were assessed using chi-square tests or Fisher’s exact tests, as appropriate. Time-to-event data, such as time to MRSA bacteremia clearance, were analyzed using Kaplan–Meier survival analysis, and treatment group differences were evaluated with the log-rank test.

An independent Data Safety Monitoring Board reviewed blinded clinical outcome data throughout the trial to ensure participant safety and maintain the integrity of the trial. The study was registered at ClinicalTrials.gov (identifier NCT05225558).

## RESULTS

### Preclinical study

In a previous study, the MIC_50_ of delpazolid was determined to be 1 µg/mL, comparable to those of vancomycin, linezolid, and daptomycin (17). The checkerboard assay results for interactions between delpazolid and either vancomycin or daptomycin are provided in Table S2. Against both the ATCC 29213 and LAC strains, the combination of delpazolid with vancomycin or daptomycin yielded an FIC index indicating indifference. In the time-kill assay, antagonism was observed when delpazolid (at 2× and 4× MIC) was combined with vancomycin at 1× MIC. However, no synergistic or antagonistic effects were observed between delpazolid and daptomycin (Fig. S1).

**Fig 2:**
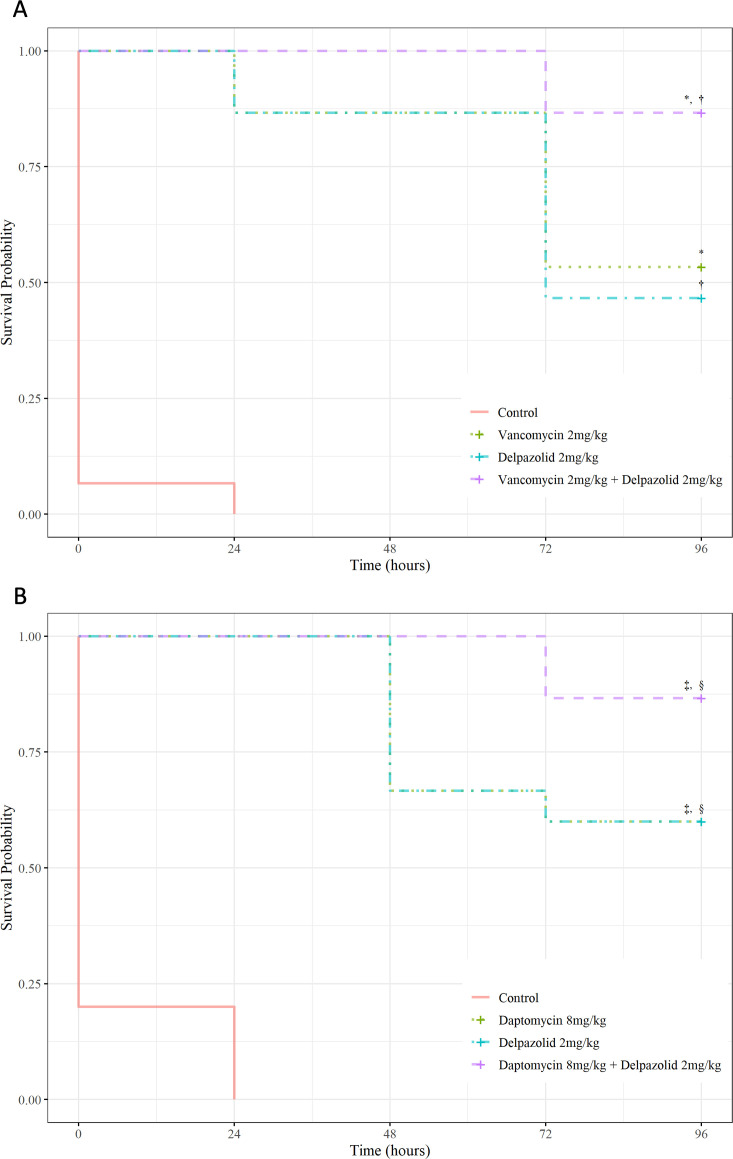
Survival curve of *Galleria mellonella* larvae infected with the MRSA LAC strain and subsequently treated with delpazolid and either (**A**) vancomycin or (**B**) daptomycin Kaplan–Meier survival analysis was performed, and survival was compared using the log-rank test. *P = 0.043, vancomycin vs vancomycin–delpazolid combination. †*P* = 0.019, delpazolid vs vancomycin–delpazolid combination. ‡*P* = 0.081, daptomycin vs daptomycin–delpazolid combination. § *P* = 0.081, delpazolid vs daptomycin–delpazolid combination. MRSA, methicillin-resistant *Staphylococcus aureus*.

In the *G. mellonella* infection model, repeated experiments showed similar results. In the representative experiment shown in Fig. 2, the combination of delpazolid and vancomycin significantly improved the survival rate compared to vancomycin alone (*P* = 0.0425) or delpazolid alone (*P* = 0.0188) (Fig. 2A). However, when delpazolid was administered with daptomycin, there was no statistically significant difference in survival compared to delpazolid alone (*P* = 0.081) or daptomycin alone (*P* = 0.081) (Fig. 2B).

### Clinical study

Participants were recruited between 26 April 2022 and 18 March 2024. The clinical trial was terminated early due to significantly low enrollment rate at the trial site, which was deemed likely to hinder the planned recruitment schedule, primarily as a result of the ongoing health crisis in Korea (18).

As shown in Fig. 1, 40 patients were randomized, and 38 received at least one dose of the study medication (safety set). The demographic and clinical characteristics of all treated patients are presented in Table 1. The mean age was 66.8 years, and half of the participants were male. Diabetes mellitus and end-stage renal disease were common in both groups. The source of bacteremia was identified in most patients, with an intravenous line being the most frequent source. Skin and soft tissue infections were more prevalent in the vancomycin group, whereas pleuropulmonary infections and infectious spondylitis were more common in the group receiving the combination of delpazolid and vancomycin. Among patients with eradicable foci, as defined by Kim et al. (19), approximately half underwent primary lesion removal. Of the randomized patients, four discontinued the study drug due to MSSA infection (*n* = 3) or lack of additional clinical evaluation (*n* = 1). Consequently, 34 patients were included in the FAS for the intention-to-treat analysis (Fig. 1), and 25 patients were assigned to the PPS group. A total of 23 patients underwent an EOT visit, and 15 completed the TOC visit. Study visits and reasons for early discontinuation of the study drug are shown in Table S3.

**TABLE 1:** Demographic and clinical characteristics of all treated patients (safety set)^*d*^

	Vancomycin + Placebo (*n* = 20)	Vancomycin + Delpazolid (*n* = 18)	Total (*n* = 38)
Age in years, mean ± SD	64.5 ± 16.47	69.3 ± 9.68	66.8 ± 13.73
≥65 years	10 (50%)	13 (72.2%)	23 (60.5%)
Sex, men	10 (50%)	11 (61.1%)	21 (55.3%)
BMI (kg/m^2^) (mean ± SD)	24.2 (4.67)	24.0 (3.18)	24.1 (3.98)
Comorbidity^*a*^	20 (100%)	18 (100%)	38 (100%)
Cardiac diseases	6 (30%)	10 (55.7%)	16 (42.1%)
Diabetes mellitus	12 (60%)	8 (44.4%)	20 (53%)
End stage renal disease	7 (35%)	7 (38.9%)	14 (36.8%)
Liver cirrhosis	1 (5%)	2 (11.1%)	3 (7.9%)
Organ transplantation	0	1 (5.6%)	1 (2.6%)
Primary site of bacteremia^*a*^			
Unknown	0	2 (11.1%)	2 (5.3%)
Skin and Soft tissue infection	6 (30%)	1 (5.7%)	7 (18.4%)
Native osteroarticular	1 (5%)	2 (11.1%)	3 (7.9%)
Intravenous line related	7 (35%)	2 (11.1%)	9 (23.7%)
Pleuropulmonary infection	0	3 (16.7%)	3 (7.9%)
Device related	1 (5%)	0	1 (2.6%)
Infective endocarditis	0	2 (11.1%)	2 (5.3%)
Infectious spondylitis	1 (5%)	2 (11.1%)	3 (7.9%)
Other	5 (25%)	4 (22.2%)	9 (23.7%)
Primary foci of infections^*b*^			
Noneradicble foci	9 (45%)	11 (61.1%)	20 (52.6%)
Eradicable foci	11 (55%)	7 (38.9%)	18 (47.4%)
Eradicated^*c*^	6 (54.6%)	4 (57.1%)	10 (55.6%)
Not eradicated	5 (45.5%)	3 (42.9%)	8 (44.4%)

^*a*^ Multiple counts were possible.

^*b*^ Primary foci of infections were divided into eradicable and noneradicable foci. Eradicable foci included surgically removable infections, drainable abscesses, and indwelling foreign bodies.

^*c*^ Among the eradicable foci, eradicated foci included those in which abscesses and indwelling foreign bodies had been drained or removed.

^*d*^ SD, standard deviation; BMI, body mass index.

**Fig 3:**
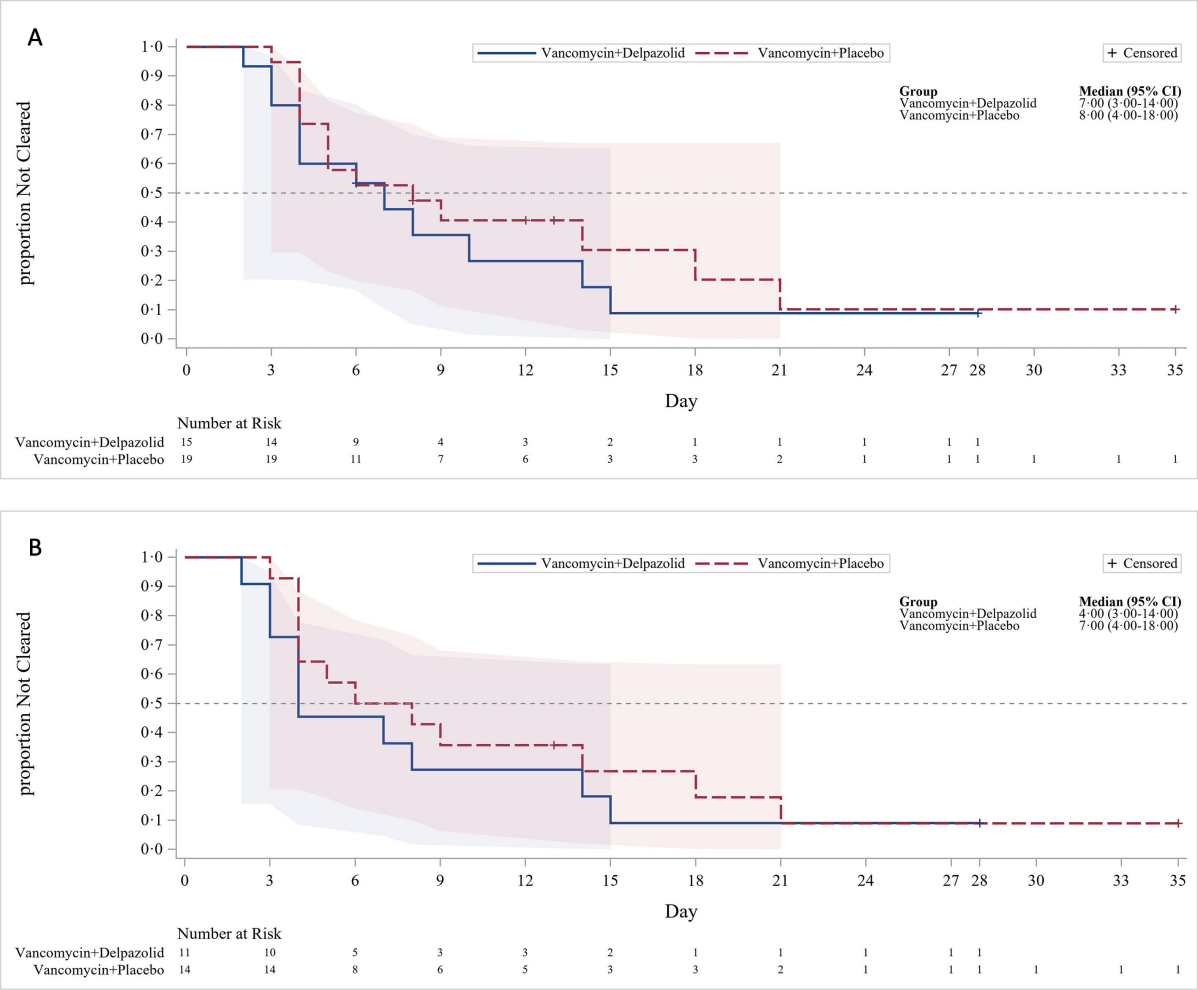
Time to clearance of MRSA bacteraemia. (**A**) In the FAS population, the median time to MRSA bacteraemia clearance was 7 days in the combination group and 8 days in the vancomycin group (*P* = 0.4538). (**B**) In the PPS population, the median time to MRSA bacteraemia clearance was 4 days in the combination group and 7 days in the vancomycin group (*P* = 0.4530). MRSA, methicillin-resistant *Staphylococcus aureus*; FAS, full analysis set; PPS, per protocol set.

In the FAS population, the overall cure rate was 9 of 15 patients (60%; 95% CI, 32.3–83.7) in the combination group (delpazolid plus vancomycin) and 10 of 19 patients (52.6%; 95% CI, 28.9–75.6) in the vancomycin group (*P* = 0.6675). In the PPS population, the overall cure rate was 9 of 11 (81.8%) in the combination group and 9 of 14 (64.3%) in the vancomycin group, consistent with the FAS population, and the difference was not statistically significant (Table 2). The overall cure rate at EOT was also higher in the combination group for both the FAS and PPS populations, with no MRSA bacteremia-attributable mortality observed in either group at EOT. The rates of persistent SAB at each time point showed no significant differences in the FAS and PPS populations. The median time to bacteremia clearance was numerically shorter in the combination group than in the vancomycin group (7 vs 8 days in the FAS population, [*P* = 0.4538]; 4 vs 7 days in the PPS population [*P* = 0.4530]), but the difference was not statistically significant (Fig. 3).

**TABLE 2:** Cure rates, MRSA bacteremia clearance rates, mortality, and relapse rate in the full analysis sets (FAS) and per protocol analysis sets (PPS)^*d*^

	Vancomycin + Placebo, *n*/*N* (%)	Vancomycin + Delpazolid, *n*/*N* (%)	95% CI for the difference	*P*-Value^*a*^
Full analysis set	19	15		
Overall cure rate^*b*^	10/19 (52.6%)	9/15 (60%)	(−26.1, 40.8)	0.6675
Overall cure at EOT	14/19 (73.7%)	12/15 (80%)		
Relapse rate at TOC	1/17 (5.9%)	2/15 (13.3%)		
Persistent SAB rate^*c*^				
Day 3	12/18 (66.7%)	6/14 (42.9%)		
Day 5	7/18 (38.9%)	4/13 (30.8%)		
Day 7	3/17 (17.7%)	1/13 (7.7%)		
Day 14	2/14 (14.3%)	0/11		
Per protocol analysis set	14	11		
Overall cure rate^*b*^	9/14 (64.3%)	9/11 (81.8%)	(−16.4, 51.4)	0.4065
Overall cure at EOT	12/14 (85.7%)	10/11 (90.9%)		
Relapse rate at TOC	1/13 (7.7%)	2/11 (18.2%)		
Persistent SAB rate^*c*^				
Day 3	9/14 (64.3%)	5/10 (50%)		
Day 5	6/14 (42.9%)	3/11 (27.3%)		
Day 7	3/14 (21.4%)	0/11		
Day 14	2/13 (15.4%)	0/11		

^*a*^ Pearson’s chi-square test or Fisher’s exact test.

^*b*^ Composite response rate means overall cure at Day 14 after the initiation of treatment.

^*c*^ The proportions of persistent bacteremia at each time point were calculated only among patients with available blood culture results at that time point.

^*d*^ MRSA, methicillin-resistant *Staphylococcus aureus*; EOT, end of treatment; TOC, test of cure (4 weeks after the end of treatment); SAB, *Staphylococcus aureus* bacteremia; CI, confidence interval.

In the safety analysis, the mean duration of delpazolid exposure was 11.7 ± 7.73 days (Table 3). The addition of delpazolid to vancomycin did not increase the incidence or severity of AEs. No deaths due to worsening MRSA bacteremia or thrombocytopenia occurred in either group. ADRs were reported in 45% of patients in the vancomycin group and 22.2% in the combination group, with most ADRs being mild and no grade 4 or 5 events reported. One serious AE (grade 5), end-stage renal disease (ESRD), was observed in the vancomycin group but was deemed unrelated to the investigational drug. Two patients discontinued treatment due to treatment-emergent AEs: one in the combination group due to decreased consciousness and one in the vancomycin group due to infective endocarditis.

**TABLE 3:** Safety parameters in all treated patients (safety set)^*a*^

	Vancomycin + Placebo *N* = 20	Vancomycin + Delpazolid *N* = 18	Total *N* = 38
Total duration of administration (days), mean ± SD	15.5 ± 10.54	11.7 ± 7.73	
TEAEs, *n* (%)	16 (80%)	13 (72.2%)	29 (76.3%)
Adverse drug reactions (ADRs), *n* (%)	9 (45%)	4 (22.2%)	13 (34.2%)
Adverse drug reactions >5% in any treatment arm, *n* (%)			
Diarrhea	0	2 (11.1%)	2 (5.3%)
Nausea	1 (5%)	1 (5.6%)	2 (5.3%)
Pruritus	3 (15%)	1 (5.6%)	3 (7.9%)
Anemia	2 (10%)	0	2 (5.3%)
SAE, *n* (%)	1 (5%)	0	1 (2.6%)
TEAEs leading to drug withdrawal, *n* (%)	1 (5%)	1 (5.6%)	2 (5.3%)

^*a*^ SD, standard deviation; TEAE, treatment-emergent adverse events; SAE, serious adverse event.

Population PK modeling and analysis were performed on 15 participants receiving combination therapy with delpazolid and vancomycin (Table S4). Plasma delpazolid concentrations were measured using liquid chromatography-tandem mass spectrometry (LC–MS/MS). Among the participants, seven had ESRD (six on hemodialysis [HD]), two had liver cirrhosis, and one had a history of liver transplantation. A two-compartment model incorporating HD status and albumin levels as covariates was selected. The mean AUC_0–*t*_ (area under the curve from time 0 to *t*) at steady state was similar between the non-ESRD (83,671 ng.h/mL) and ESRD groups (99,065 ng.h/mL), with comparable mean *C*_max_ values. However, participants undergoing HD during drug administration exhibited approximately 50% lower mean AUC_0–*t*_ and C_max_ compared to non-HD participants. Despite these lower plasma concentrations, all but one participant in the HD group achieved an overall cure at 14 days post-treatment.

Participants with a history of cirrhosis, liver transplantation, or moderate-to-severe liver dysfunction (per the National Cancer Institute-Organ Dysfunction Working Group criteria) were classified into the hepatic impairment (HI) group. This group had a mean AUC_0–*t*_ of 152,880 ng.h/mL, approximately three times higher than that of the no-HI group (59,842 ng.h/mL). Similarly, the mean *C*_max_ of the HI group was twice that of the no-HI group.

Among the 38 participants in the safety-set population, all baseline *S. aureus* isolates were susceptible to vancomycin and linezolid (Table S5). The MIC_50_ and MIC_90_ of delpazolid were 1 µg/mL and 2 µg/mL, respectively. In the delpazolid-vancomycin group, the vancomycin and daptomycin MIC_50_/MIC_90_ values were both 1/1 µg/mL, whereas in the vancomycin-only group, they were 0.5/1 µg/mL. Antibiotic susceptibility testing was repeated for follow-up isolates from seven patients with MRSA bacteremia and positive follow-up blood cultures. On day 14, MICs were evaluated in one participant from the delpazolid group and two from the control group, with no increases from baseline. At EOT, one participant in the treatment group also showed no increase in MIC.

## DISCUSSION

This Phase 2a exploratory study was designed to evaluate the feasibility, safety, and clinical effects of combined delpazolid with vancomycin for the treatment of MRSA bacteremia based on supportive preclinical data. *In vitro* and *in vivo* preclinical models indicated that delpazolid exhibited antimicrobial activity comparable to that of vancomycin and daptomycin. In the *G. mellonella* infection model, combination therapy significantly improved survival rates compared to monotherapy. In the early terminated clinical study, the combination regimen demonstrated an acceptable safety profile, with no apparent increase in the frequency or severity of adverse events compared with vancomycin monotherapy. While the overall cure rate and bacteremia clearance appeared numerically higher in the combination group, these differences were not significant.

Given the challenges in treating MRSA bacteremia and its high mortality rate, various combination antibiotic regimens have been explored to enhance efficacy and prevent resistance through complementary mechanisms of action. However, randomized clinical trials evaluating combination therapy for *S. aureus* bacteremia have not demonstrated improved clinical outcomes compared with standard monotherapy. Notably, the CAMERA2 trial (vancomycin or daptomycin plus β-lactam), BACSARM (daptomycin plus fosfomycin), SAFO (daptomycin plus fosfomycin for MSSA), ARREST (adjunctive rifampicin), and DASH (daptomycin plus cefazolin or cloxacillin) studies all failed to show clinical benefit and in some cases were limited by increased adverse events or early termination (20–24). A retrospective study reported that linezolid-based regimens cleared blood cultures within 72 h more effectively than continued vancomycin in cases of persistent MRSA bacteremia (25). Delpazolid, an oral oxazolidinone, exhibits favorable pharmacokinetic properties, including lower mitochondrial toxicity and reduced risks of drug-drug interactions or serotonin syndrome compared to other agents (26). A preclinical time-kill assay showed antagonism between delpazolid and vancomycin, similar to *in vitro* synergy test results observed with linezolid against certain *S. aureus* strains (27). However, discrepancies often exist between *in vitro* and *in vivo* findings. In one study, the combination of vancomycin and linezolid exhibited antagonism against a specific MRSA strain in a time-kill assay; however, this antagonism was not observed in a murine peritonitis model (28). Similarly, in our *G. mellonella* infection model, the delpazolid–vancomycin combination therapy improved survival rates, contrary to the *in vitro* results. These findings underscore the complexity of drug–drug interactions and the limitations of *in vitro* models in predicting *in vivo* outcomes. The observed preclinical data should therefore be interpreted cautiously, and additional clinical studies are warranted to clarify the translational relevance of this combination in MRSA infections.

In our clinical study, the combination therapy did not increase the frequency or severity of AEs compared to standard vancomycin monotherapy, and no cases of thrombocytopenia were observed. Additionally, no MIC creep was detected in follow-up blood culture isolates. Notably, in a separate study comparing linezolid and delpazolid for the treatment of multidrug-resistant tuberculosis, the incidence of ADRs was substantially lower with delpazolid 800 mg BID (18.8%) compared to linezolid 600 mg BID (50%) (11).

The study was terminated early due to slow enrollment, resulting in a limited sample size that precludes any definitive conclusions regarding clinical outcomes. Although numerically higher cure rates and faster bacteremia clearance were observed in the combination group, these differences were not statistically significant and should be interpreted with caution. Vancomycin treatment failures have been attributed to its slow bactericidal activity and the emergence of strains with reduced susceptibility, including those with elevated MICs or heteroresistant vancomycin-intermediate *S. aureus* (2). While the 2011 MRSA treatment guidelines define persistent bacteremia as lasting ≥7 days (29), recent reports suggest that any positive follow-up blood culture after initiating appropriate therapy should raise concern (30, 31). In clinical practice, persistent bacteremia often prompts antibiotic escalation or combination therapy (32). Given that delayed clearance of *S. aureus* bacteremia is independently associated with increased mortality and risk of metastatic complications (31), these findings highlight the need for further investigation in future Phase III trials. PK analysis suggested that delpazolid is likely removed via HD. Despite lower plasma concentrations in patients receiving HD, an overall cure was observed in all but one patient on day 14 post delpazolid administration. Although the exact elimination pathway of delpazolid remains to be elucidated, markedly higher plasma levels in patients with hepatic impairment suggest that the liver may be the primary metabolic route. These findings underscore the need for further studies to optimize delpazolid dosing strategies in specific patient populations, including those undergoing HD and those with hepatic dysfunction.

This study had certain limitations. First, due to the ongoing public health crisis in Korea (33), recruitment of clinical trial participants was challenging, ultimately leading to early termination of the study and a limited sample size. This small cohort may also have contributed to baseline imbalances in infection sources between treatment groups. Second, as delpazolid is an oral antibiotic, severely ill patients were excluded from the trial, and no mortality attributable to MRSA bacteremia was observed within the study cohort. Third, the absence of a rapid diagnostic test for MRSA bacteremia necessitated randomization within 96 h of the index blood culture in patients who had already initiated empirical vancomycin therapy within 72 h. Fourth, less than half of the enrolled participants completed the TOC visit, resulting in substantial loss to follow-up, which may have influenced the assessment of longer-term outcomes.

Although numerous studies have demonstrated the potential of combination antibiotic therapy for *S. aureus* bacteremia *in vitro* and in animal models, these findings have not been consistently replicated in prospective clinical trials assessing clinically meaningful outcomes (34). Our study adds to the growing body of evidence suggesting that delpazolid–vancomycin combination therapy may offer therapeutic benefit in this context.

In conclusion, our early-terminated phase 2a study provides that the delpazolid-vancomycin combination therapy was feasible and generally well tolerated in patients with MRSA bacteremia. These findings underscore the need for larger, adequately powered clinical trials to clarify its clinical role.

## Data Availability

De-identified participant data will be made available upon requests directed to the chief investigator H.B.K. Proposals will be reviewed and approved by the sponsor, chief investigators, and collaborators based on scientific merit. After approval of a proposal, data can be shared through a secure online platform after signing a data access agreement. Outcome data with accompanying pharmacokinetic data will be made available on reasonable request made to the corresponding author.

## SUPPLEMENTAL MATERIAL

Figure S1A spectrum.03361-25-s0001.tiff Time-kill curve of MRSA LAC strains treated with delpazolid and vancomycin.

Figure S1B spectrum.03361-25-s0002.tiff Time-kill curve of MRSA LAC strains treated with delpazolid and daptomycin.

Supplemental material spectrum.03361-25-s0003.docx Tables S1 to S5 and legend for Fig. S1.

ASM does not own the copyrights to Supplemental Material that may be linked to, or accessed through, an article. The authors have granted ASM a non-exclusive, world-wide license to publish the Supplemental Material files. Please contact the corresponding author directly for reuse.
