# Supplementary figures and images for: Preclinical and clinical evaluation of vancomycin plus delpazolid combination therapy for MRSA bacteremia: a multicenter, double-blinded, randomized, parallel design, phase IIa clinical trial
Source: Microbiol Spectr. 2026 Feb 18;14(4):e03361-25. doi: 10.1128/spectrum.03361-25 (PMC13055365; doi:10.1128/spectrum.03361-25)

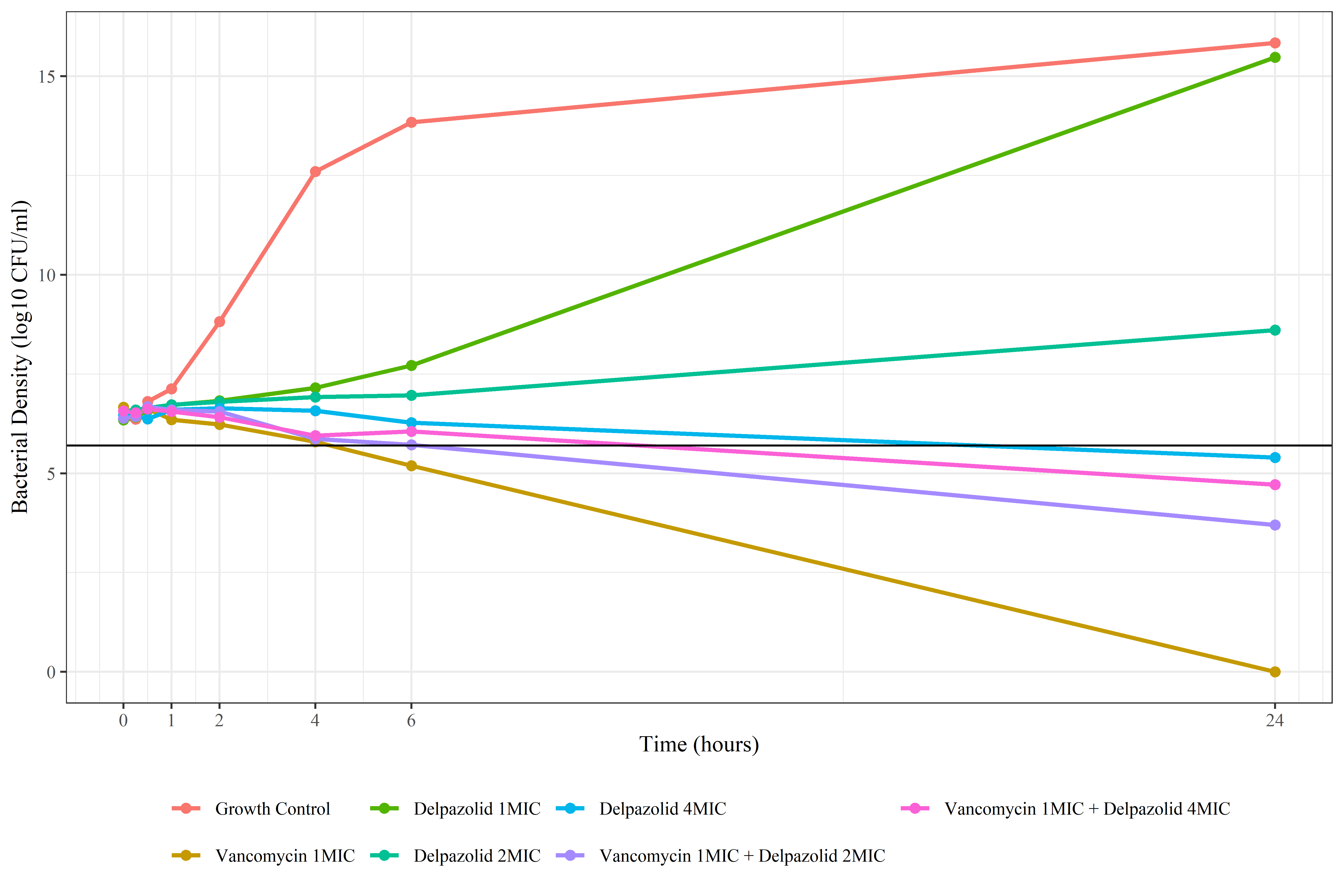

Supplement: Figure S1A — Time-kill curve of MRSA LAC strains treated with delpazolid and vancomycin. [file spectrum.03361-25-s0001.tiff]

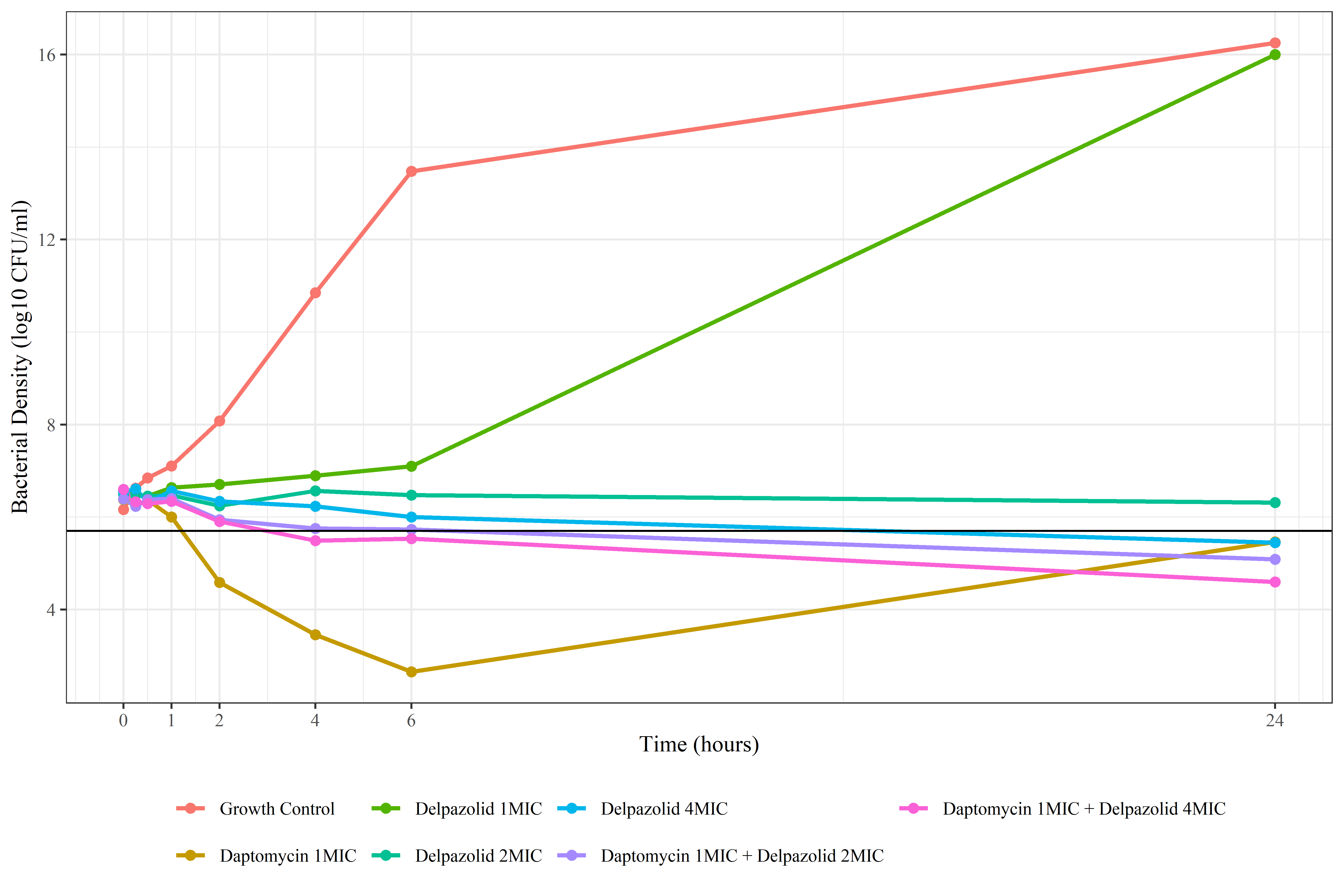

Supplement: Figure S1B — Time-kill curve of MRSA LAC strains treated with delpazolid and daptomycin. [file spectrum.03361-25-s0002.tiff]
