# Supplementary material for: Preclinical and clinical evaluation of vancomycin plus delpazolid combination therapy for MRSA bacteremia: a multicenter, double-blinded, randomized, parallel design, phase IIa clinical trial
Source: Microbiol Spectr. 2026 Feb 18;14(4):e03361-25. doi: 10.1128/spectrum.03361-25 (PMC13055365; doi:10.1128/spectrum.03361-25)
Supplement: Supplemental material — Tables S1 to S5 and legend for Fig. S1. [file spectrum.03361-25-s0003.docx]

**Supplementary Materials**

**Table S1. Definitions of outcome**

| **Outcome** | **Definition** |
| --- | --- |
| Clinical improvement | Resolution of infection-related symptoms present at the time of study enrolment, with no evidence of new infections or secondary infections caused by MRSA. The treating clinician at each site evaluated clinical improvement according to these predefined criteria. |
| Clinical failure | Participants determined to have failed clinical treatment during the treatment period are considered to have failed treatment in all subsequent evaluations.  Clinical failure is defined by any of the following criteria: ✓ death due to MRSA bacteremia during the treatment period. ✓ requirement for alternative antibiotic therapy for the treatment of MRSA bacteremia (note: if vancomycin treatment fails during the treatment period, a switch to daptomycin is allowed based on investigator judgment. Daptomycin may be followed by oral antibiotics, excluding the oxazolidinone class, after a minimum of 14 days of vancomycin (or daptomycin) treatment. Planned visits must continue after any antibiotic change.). ✓ emergence of a new infection or secondary infection caused by MRSA requiring additional treatment. |
| Clearance of MRSA bacteremia | Two consecutive negative blood cultures for MRSA.  If the first negative blood culture was identified, an additional blood culture was required within 3 days of the initial result. |
| Persistent MRSA bacteremia | Persistent positivity for MRSA in blood cultures. |
| Time (in days) to achieve clearance of MRSA bacteremia. | From the date of the first blood culture, MRSA positivity to the date of the first blood culture showing a negative result |
| MRSA bacteremia relapse | Recurrence of MRSA bacteremia after two consecutive negative blood cultures occurring before the Test of Cure (TOC) visit. |

MRSA, methicillin-resistant *Staphylococcus aureus*

**Table S2. Checkerboard assay results for delpazolid in combination with vancomycin or daptomycin against *Staphylococcus aureus* isolates**

|  |  | **ATCC 29213**  **(MSSA)** | **LAC**  **(MRSA)** |
| --- | --- | --- | --- |
| MIC | Delpazolid | 2 | 1 |
|  | Vancomycin | 0.5 | 0.5 |
|  | Daptomycin | 0.5 | 0.5 |
|  | Delpazolid/vancomycin | 2/0.125 | 1/0.125 |
|  | Delpazolid/daptomycin | 2/0.125 | 1/0.125 |
| Lowest FIC index  (interpretation) | Delpazolid/vancomycin | 2.25 (indifference) | 1.25 (indifference) |
|  | Delpazolid/daptomycin | 2.25 (indifference) | 1.25 (indifference) |

MIC; minimum inhibitory concentrations, FIC; fractional inhibitory concentration, ATCC; American Type Culture Collection (Manassas, VA, United States of America), LAC; Los Angeles County, MRSA; methicillin-resistant *S. aureus*, MSSA; methicillin-susceptible *S. aureus*

**Table S3. Study visits and reasons for early discontinuation of study medication**

|  | **Vancomycin+placebo**  **N=20, n (%)** | **Vancomycin+Delpazolid**  **N=18, n (%)** | **Total N=38,n (%)** |
| --- | --- | --- | --- |
| Completed day 14 visit of therapy | 14 (70%) | 11(61%) | 25 (66%) |
| Completed EOT | 13 (65%) | 10 (56%) | 23 (61%) |
| Completed TOC visit | 9 (55%) | 6 (33%) | 15 (39%) |
| Discontinued study drugs early | 6 (30%) | 7 (39%) | 13 (34%) |
| Reason for early study drugs discontinuation |  |  |  |
| Continuation criteria not met | 1 | 3 | 4 |
| Consent withdrawal | 3 | 3 | 6 |
| Treatment failure | 1 | 0 | 1 |
| Adverse event | 0 | 1 | 1 |
| Others | 1 | 0 | 1 |

N, number; EOT, end of treatment; TOC, test of cure (four weeks after the end of treatment)

**Table S4. Delpazolid pharmacokinetic parameters by population PK modelling**

|  | **No ESRD** | | **ESRD** | | **Not undergoing HD**  **(No ESRD/ESRD without HD receiving as medical history)** | | **Undergoing HD**  **(ESRD with HD receiving as medical history)** | | **No HI/LC** | | **HI/LC** | | **Total** | |
| --- | --- | --- | --- | --- | --- | --- | --- | --- | --- | --- | --- | --- | --- | --- |
|  | **n=8** | | **n=7** | | **n=9** | | **n=6** | | **n=10** | | **n=5** | | **N=15** | |
|  | Mean | SD | Mean | SD | Mean | SD | Mean | SD | Mean | SD | Mean | SD | Mean | SD |
|  |  |  |  |  |  |  |  |  |  |  |  |  |  |  |
| AUC_0-_t, ss (ng·h/mL) | 83,671 | 75,411 | 99,065 | 107,966 | 112,462 | 111,518 | 58,444 | 11,292 | 59,842 | 26,423 | 152,880 | 137,383 | 90,855 | 88,895 |
| C_max_, ss (ng·h/mL) | 8,610 | 5,715 | 8,577 | 9,156 | 10,903 | 8,712 | 5,132 | 938 | 6,216 | 1,624 | 13,353 | 11,598 | 8,595 | 7,229 |
| C_min_, ss (ng·h/mL) | 5,541 | 6,209 | 7,781 | 8,644 | 7,955 | 9,284 | 4,533 | 1,032 | 3,992 | 2,331 | 11,774 | 11,026 | 6,586 | 7,255 |
| T_max_, ss (h) | 3.083 | 1.515 | 4.110 | 0.861 | 3.286 | 1.542 | 3.978 | 0.861 | 3.291 | 1.429 | 4.105 | 0.984 | 3.562 | 1.322 |
| t1/2β (h) | 1,689 | 4,369 | 17,980 | 41,721 | 13,973 | 37,076 | 2,270 | 3,949 | 425 | 572 | 27,025 | 47,976 | 9,292 | 28,745 |
| MRT, ss (h) | 1,159 | 3,024 | 13,340 | 28,912 | 9,730 | 25,868 | 2,513 | 4,304 | 436 | 654 | 19,657 | 33,160 | 6,843 | 20,060 |

AUC, area under the curve; C, concentration; C_max_, maximum plasma concentration; ESRD, end-stage renal disease; HD, hemodialysis; HI, hepatic impairment; LC, liver cirrhosis; MRSA, methicillin-resistant *Staphylococcus aureus*; MSSA, methicillin-susceptible *S. aureus*; MRT, mean residence time; PK, pharmacokinetic; T_max_, time to reach C_max_; SD, standard deviation; SS, steady-state

Hepatic impairment was defined as a history of cirrhosis, liver transplantation, or moderate-to-severe liver dysfunction (according to the NCI-ODWG criteria).

**Table S5. *In vitro* activity of delpazolid and comparator antimicrobial agents against 38 MRSA first blood isolates**

| Antimicrobials | N (cumulative %) of isolates inhibited at MIC (µg/mL) of: | | | | | MIC_50_ | MIC_90_ | % susceptible | % resistant |
| --- | --- | --- | --- | --- | --- | --- | --- | --- | --- |
|  | ≤0.25 | 0.5 | 1 | 2 | >2 |  |  |  |  |
| Vancomycin | 0 (0) | 16(42) | 22(58) | 0 (0) | 0 (0) | 1 | 1 | 100 | 0 |
| Daptomycin | 0 (0) | 9 (24) | 27(71) | 2 (5) | 0 (0) | 1 | 1 | 95 | 5 |
| Linezolid | 0 (0) | 0 (0) | 6 (16) | 30 (79) | 2 (5) | 2 | 2 | 100 | 0 |
| Delpazolid | 0 (0) | 1 (4) | 32 (84) | 5 (13) | 0 (0) | 1 | 2 | N/A | N/A |

MRSA, methicillin-resistant *Staphylococcus aureus*; MIC, minimum inhibitory concentration N: number; N/A: not applicable

**Supplementary Figure legends**

**Fig. S1**. Time-kill curves of MRSA LAC strains treated with delpazolid and either vancomycin or daptomycin (**A**) Time-kill curve of MRSA LAC strains treated with delpazolid and vancomycin

(**B**) Time-kill curve of MRSA LAC strains treated with delpazolid and daptomycin.

Bacterial density was counted at 15 min, 30 min, 1, 2, 4, 6, and 24 h after culture.

MRSA, methicillin-resistant *Staphylococcus aureus*
